# Ameliorative effects of propolis and wheat germ oil on acute toxoplasmosis in experimentally infected mice are associated with reduction in parasite burden and restoration of histopathological changes in the brain, uterus, and kidney

**DOI:** 10.3389/fvets.2024.1357947

**Published:** 2024-03-01

**Authors:** Ehab Kotb Elmahallawy, Fatma Abo Zakaib Ali, Enrique Raya-Álvarez, Alaa Fehaid, Khaled A. Abd El-Razik, Hassan Ali Mohamed El Fadaly, Manal F. El-Khadragy, Amal S. M. Sayed, Ashraf H. Soror, Alaa S. Alhegaili, Amira A. Saleh, Abdulsalam A. M. Alkhaldi, Abd El-Nasser A. Madboli, Ahmad Agil, Ashraf Mohamed Barakat

**Affiliations:** ^1^Departamento de Sanidad Animal, Grupo de Investigación en Sanidad Animal y Zoonosis (GISAZ), Facultad de Veterinaria, Universidad de Córdoba, Córdoba, Spain; ^2^Department of Zoonoses, Faculty of Veterinary Medicine, Sohag University, Sohag, Egypt; ^3^Department of Pathology and Clinical Pathology, Faculty of Veterinary Medicine, Sohag University, Sohag, Egypt; ^4^Rheumatology Department, Hospital Universitario San Cecilio, Av. de la Investigación s/n, Granada, Spain; ^5^Forensic Medicine and Toxicology Department, Faculty of Veterinary Medicine, Mansoura University, Mansoura, Egypt; ^6^Department of Animal Reproduction, Veterinary Research Institute, National Research Centre, Giza, Egypt; ^7^Department of Zoonotic Diseases, National Research Centre, Giza, Egypt; ^8^Department of Biology, College of Science, Princess Nourah bint Abdulrahman University, Riyadh, Saudi Arabia; ^9^Department of Zoonoses, Faculty of Veterinary Medicine, Assiut University, Asyut, Egypt; ^10^Department of Medical Laboratory, College of Applied Medical Sciences, Prince Sattam bin Abdulaziz University, Alkharj, Saudi Arabia; ^11^Department of Medical Parasitology, Faculty of Medicine, Zagazig University, Zagazig, Egypt; ^12^Biology Department, College of Science, Jouf University, Sakaka, Saudi Arabia; ^13^Department of Pharmacology, Biohealth Institute Granada (IBs Granada) and Neuroscience Institute, School of Medicine, University of Granada, Granada, Spain

**Keywords:** propolis, WGO, ameliorate, acute, toxoplasmosis, real time-PCR, histopathological changes

## Abstract

Toxoplasmosis continues to be a prevalent parasitic zoonosis with a global distribution. This disease is caused by an intracellular parasite known as *Toxoplasma gondii*, and the development of effective novel drug targets to combat it is imperative. There is limited information available on the potential advantages of wheat germ oil (WGO) and propolis, both individually and in combination, against the acute phase of toxoplasmosis. In this study, acute toxoplasmosis was induced in Swiss albino mice, followed by the treatment of infected animals with WGO and propolis, either separately or in combination. After 10 days of experimental infection and treatment, mice from all groups were sacrificed, and their brains, uteri, and kidneys were excised for histopathological assessment. Additionally, the average parasite load in the brain was determined through parasitological assessment, and quantification of the parasite was performed using Real-Time Polymerase Chain Reaction targeting gene amplification. Remarkably, the study found that treating infected animals with wheat germ oil and propolis significantly reduced the parasite load compared to the control group that was infected but not treated. Moreover, the group treated with a combination of wheat germ oil and propolis exhibited a markedly greater reduction in parasitic load compared to the other groups. Similarly, the combination treatment effectively restored the histopathological changes observed in the brain, uterus, and kidney, and the scoring of these reported lesions confirmed these findings. In summary, the present results reveal intriguing insights into the potential therapeutic benefits of wheat germ oil and propolis in the treatment of acute toxoplasmosis.

## 1 Introduction

Toxoplasmosis is a well-known zoonotic infection that is caused by an obligate intracellular parasite named *Toxoplasma Gondii* (*T. gondii*). It is one of the most important parasites that affects the public health because of the involvement of warm-blooded animals and humans as an intermediate host of *T. gondii*. This foodborne disease is transmitted mainly by ingestion of either water and vegetables contaminated with oocytes after their shedding from the infected feline (cats) as a definitive host, or uncooked meat containing the tissue cyst (1–3). The other less common routes of transmission include the infected organ transplantation (4) and vertical transmission during pregnancy from the infected mother to the unborn child via placenta (5). The life cycle of *T. gondii* has three stages of development including the tachyzoite stage that is characteristic in case of acute infection because of its rapid multiplication, bradyzoite stages that is characteristic in case of chronic infection and its slow multiplication and its existence as muscular or brain tissues cysts, and lastly the sporozoite stage of the definitive hosts (Feline species) which is released in the oocysts with feces deposition (6). Generally, toxoplasmosis is asymptomatic in case of people or animals with potent immune system, or it may induce non-specific or flu-like symptoms (7, 8). However, the disease could be manifested with severe symptoms or even fatal in immunocompromised hosts (9).

Taken into account, acute toxoplasmosis in people with weak immune system can induce variable degrees of clinical signs ranging from mild to severe symptoms. Headache, incoordination, seizures, and severe inflammation of the retina (ocular toxoplasmosis) are the most common symptoms. Severe cases of toxoplasmosis usually accompanied by swollen lymph nodes, brain damage, and encephalitis (10). Congenital toxoplasmosis which transmitted during the pregnancy leads to fetal death and abortion, if not, infant is commonly suffered from hydrocephalus, chorioretinitis and sometimes ended by encephalopathy and blindness (11). Beside the infection’s effect on the central nervous system, many histopathological and biochemical changes were also reported in previous studies in different organs including liver, kidney, uterus and brain (12, 13).

The typical treatment of toxoplasmosis is a combination of pyrimethamine and sulfadiazine as two different antimicrobials which inhibit the dihydrofolate reductase (DHFR) and block the folic acid synthesis, respectively (14). As a consequence of the acute infection, the active tachyzoites encyst and become bradyzoites in different tissues and remain idle in people with potent immune system, except the cysts in the retina which cause recurrent chorioretinitis after frequent activation (14). Unfortunately, the commonly used drugs are only effective against the tachyzoites but not bradyzoites cysts (15). Also, many used treatment protocols are required monitoring different vital parameters such as blood count, hepatic, and renal markers to avoid their side effects specially in the immunocompromised patients (9). Therefore, investigating novel effective therapies is very important recently. Medicinal plants-based therapies are highly targeted by many researchers nowadays to get the desirable effects with no or minimal side effects in relative to the commonly used chemical drugs. Many parasitic infections, such as coccidiosis, leishmaniasis, and toxoplasmosis, were reported to be treated by plant-based compounds including oregano, garlic, mushrooms, and citric fruit’s extracts (16–18). Propolis is a honeybee product, which is commonly used in medical applications showing its antimicrobial, anti-inflammatory, antioxidant, and anticancer properties (19). Moreover, propolis could inhibit the *Trypanosomes* growth (16), and suppress the *T. gondii* tachyzoites development (20), along with an inhibitory action against different intracellular and extracellular protozoan parasites (21). Wheat (*Triticum aestivum*) is an edible whole grain which is highly consumed all over the world because of its various benefits (22). Wheat contains 5% germ which is a good source of antioxidants and amino acids, therefore, it has been used as a nutrient supplementation (23, 24). Interestingly, WGO contains tocopherol derivatives, n-3 fatty acids, fat-soluble carotenoids, and phenolic compounds (25–27). It has been reported in many *in vitro* and *in vivo* studies for its ability to reduce the oxidative stress, improve the lipid metabolism, enhance the fertility, and inhibit the carcinogenesis (25, 26, 28–30). Owing to the active ingredients of both propolis and WGO, they are commonly used as herbal therapies in many diseased conditions, including chronic toxoplasmosis (31–34). However, their potential protective effect against acute toxoplasmosis is not clear yet. Thus, the aim of this study was to investigate the anti-parasitic role of combination of propolis and WGO in treating acute toxoplasmosis in mice, as an herbal therapy. This would help to develop a new plant-based anti-parasitic drug with minimal side effects.

## 2 Materials and methods

### 2.1 Substances

Propolis and WGO were available commercially in Local Herbs Company, Cairo, Egypt. All used substances were obtained from Cairo, Egypt with an analytical grade quality. Characterization and identification of both herbal substances was performed using GC–MS analysis of silylated metabolites as mentioned in previous works (31–33, 35–37).

### 2.2 Parasite (*Toxoplasma Gondii*) material and parasite preparation

Zoonoses department in National Research Center (NRC), Egypt, provided us with tachyzoites of RH virulent strain of *T. gondii*. Briefly, the parasitic material was intraperitoneally injected into mice via serial passages for maintenance. Five days later, tachyzoites were collected from the peritoneal cavity and parasites were counted by hemocytometer (38). Then, at age of 7 weeks, experimental mice were infected with 1 mL saline containing 3.5 × 10^3^ parasitic cysts and considered as the infected mice for this experiment as described elsewhere (39, 40).

### 2.3 Experimental protocol

**Figure 1:**
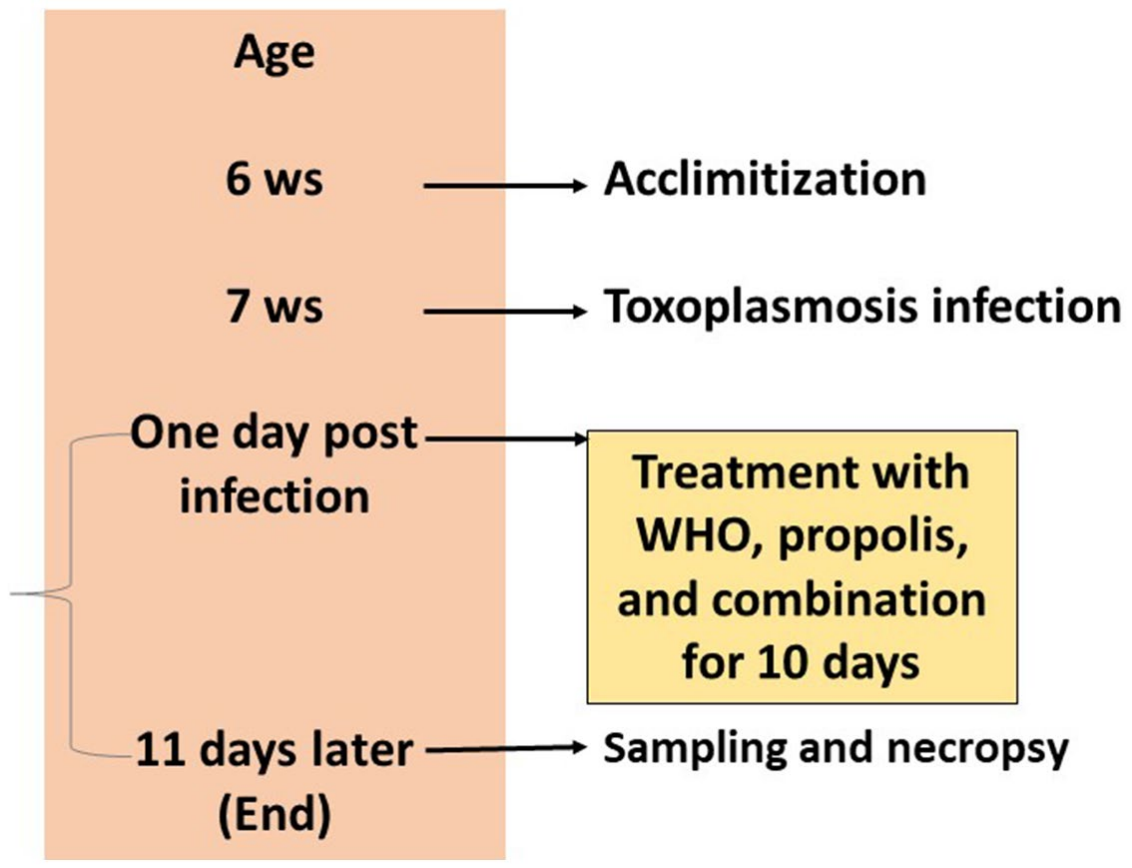
Timeline of the experimental protocol explaining the age of mice in weeks, treatments, and sampling.

Fifty female Swiss albino mice with a weight of 30–35 g and age of 6 weeks were acclimatized for 1 week in the experimental environment. Housing followed the standard conditions in well-ventilated clean cages at a temperature of 25°C, and 12 h light/12 h dark cycles. Mice were fed with standard pellets and water was allowed *ad libitum*. Ten mice were used as a negative control group (G1) which is not infected and not treated as well. The rest of mice were infected at age of 7 weeks as mentioned before and were randomly divided into 4 groups with 10 mice each. The positive control group (G2) served as the infected and non-treated group. The other four groups were infected and treated with different treatments for 10 days starting the next day of the infection as shown in illustration 1. The third group (G3) was infected and orally treated with 0.1 mL/day propolis extract (31–33). The fourth group (G4) was infected and orally treated orally with 0.2 mg/kg/day WGO in 1.5 mL PBS (41). The last group (G5) was infected and orally treated with both propolis and WGO extract using the same doses for G3 and G4. During the experimental period, health condition and clinical signs of mice were recorded regularly for the presence of any other abnormalities and fecal specimens were analyzed for the existence of parasitic cysts (42, 43). The experimental protocol is shown in Figure 1.

### 2.4 Samples collection for different analysis

At the end of the experiment, sacrificing of mice was done and samples from brains’ tissues were collected for parasitological analysis. Brain tissues were suspended and homogenized in saline, then 0.1 mL suspension were smeared on a glass slide for tissue cysts count using 10 high-power fields (HPF). Cysts count was calculated following a previously described method (44). For the Real-Time Polymerase Chain Reaction (RT-PCR), specimens of brain tissues (25 mg) were collected and washed thoroughly with sterile PBS buffer, and then DNA was extracted using tissue DNA extraction kit following the manual instructions. Samples were kept for further RT-PCR reaction. For histopathological examination, specimens from brain, uterus and kidney were collected and kept in 10% neutral buffered formalin.

### 2.5 RT-PCR analysis

Taq qPCR Green Master Mix I (Cat no: QLMM12, Vivantis Co., Malysia) was used for RT-PCR reaction. The used primer set of targeted *Toxoplasma* P29 gene was Q-f: CAGCATGGATAAGGCATCTG, and Q-r: GTTGCTCCTCTGTTAGTTCC. RT-PCR analysis was performed using MX30005P Agilent Real-Time PCR detection system (Germany) and the thermocycling conditions were 95°C for 2 min, followed by 40 cycles of 95°C for 15 s and 60°C for 20 s. Results were expressed as a change fold of cycles threshold (Ct) compared to the control samples’ threshold. High Ct values represent low load of *Toxoplasma* and vice versa.

### 2.6 Histopathological examination and scoring

The previously formalin-preserved tissues’ specimens were processed and embedded in paraffin wax to get the blocks. Sections of 5-micron thickness were sectioned from all blocks, stained with hematoxylin and eosin (H&E) (45) and examined microscopically for any pathological alteration. For histopathological scoring, 10 sections per experimented group at least were inspected by a pathologist and photos were taken with a magnification lens of 40x (46). In addition, cysts counts were recorded in 10 different areas (47). The interpretation of the scores followed a previous scoring method (48) and was as follows: score (4) means marked alteration in which more than 40% of tissues were affected, score (3) means moderate alteration in which 21–40% of tissues were affected, score (2) means mild alteration in which 11–20% of tissues were affected, and score (1) is the minimal score in which 0–10% of tissues were affected.

### 2.7 Statistical analysis

Data were statistically analyzed using a statistical software program (SPSS 15.0). The differences between the mean values of experimental groups were determined using one-way ANOVA with Tukey’s *post-hoc* multiple comparison tests. The *p* value less than 0.05 was considered to be statistically significant. Data were presented as mean ± standard deviation (SD).

## 3 Results

### 3.1 Parasitological assessment

**Figure 2:**
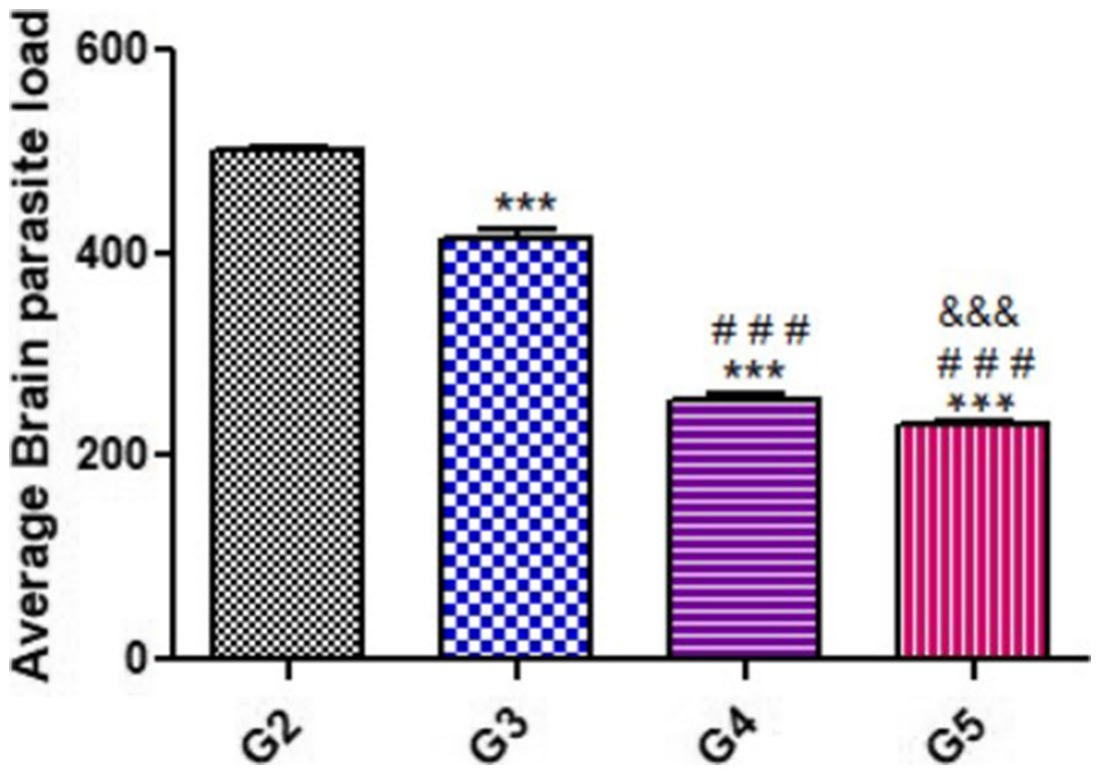
Average Brain parasite load (ABPL) of treated mice as compared with untreated mice during acute phase (14 DPI). Significant differences (G2 vs. other groups are marked by asterisks), (G3 vs. G4 and G5 are marked by #), (G4 vs. G5 are marked by &) all through one-way ANOVA with Tukey’s *post-hoc* test: ***, ###, && *p* ≤ 0.001.

As shown in Figure 2, the average brain parasitic loads (ABPL) were significantly reduced in groups treated with propolis and WGO (G3, G4, and G5), when compared to the untreated infected mice (G2).

**Table 1:** The CT values and absolute quantity of both the standard and the treated samples.

NTC	G1	NTC (non-infected- non treated)	No Ct	No Ct
Positive Control	G2-a	Standard infected non-treated	22.14	7.60E-03
	G2-b	Standard infected non-treated	22.30	7.60E-01
	G2-c	Standard infected non-treated	26.11	7.60E-05
	G2-d	Standard infected non-treated	23.27	7.50E-05
	G2-e	Standard infected non-treated	22.91	7.60E-02
Treated	G3-a	Infected Treated with Propolis	25.52	6.5E-1
	G3-b	Infected Treated with Propolis	27.11	6.1E-1
	G3-c	Infected Treated with Propolis	27.37	7.2E-1
	G4-d	Infected Treated with Propolis	26.45	6.5E-2
	G4-e	Infected Treated with Propolis	25.21	6.9E-1
	G4-a	Infected Treated with Wheat germ oil	32.55	4.4E-1
	G4-b	Infected Treated with Wheat germ oil	30.54	5.1E-2
	G4-c	Infected Treated with Wheat germ oil	31.58	5.9E-1
	G4-d	Infected Treated with Wheat germ oil	30.30	5.3E-3
	G4-e	Infected Treated with Wheat germ oil	31.67	4.9E-3
	G5-a	Infected Treated with combination of Wheat germ oil and Propolis.	35.31	3.6E-2
	G5-b	Infected Treated with combination of Wheat germ oil and Propolis.	34.10	4E-2
	G5-c	Infected Treated with combination of Wheat germ oil and Propolis.	36.22	4.4E-2
	G5-d	Infected Treated with combination of Wheat germ oil and Propolis.	36.17	3.6E-1
	G5-e	Infected Treated with combination of Wheat germ oil and Propolis.	35.82	3.6E-3

### 3.2 *Toxoplasma* P29 gene assessment

In the present work, *Toxoplasma* P29 gene quantification was significantly high in the control positive group (G2) as shown in Table 1. Furthermore, all tested samples gave positive results with clear variation in the product quantities as clarified in Table 2. The significant lowest *Toxoplasma* P29 gene quantification was observed in (G5) which treated with both propolis and WGO together. These findings suggested the promising effect of herbal treatment against the *Toxoplasma* P29 gene elevations after the infection.

**Table 2:** The variable change in the *Toxoplasma gondii* load after treatment with the herbal substances in comparison with the control drug, infected, non-treated animals.

Sample ID	Quantity
C (G2; Infected untreated) sample	0.00E+00
S1 (G3; Treated with Propolis)	6.64E-1
S2 (G4; Treated with Wheat germ oil)	5.12E-2
S3 (G5: Treated with combination of Wheat germ oil and Propolis)	3.84E-2

### 3.3 Histopathological changes

**Figure 3:**
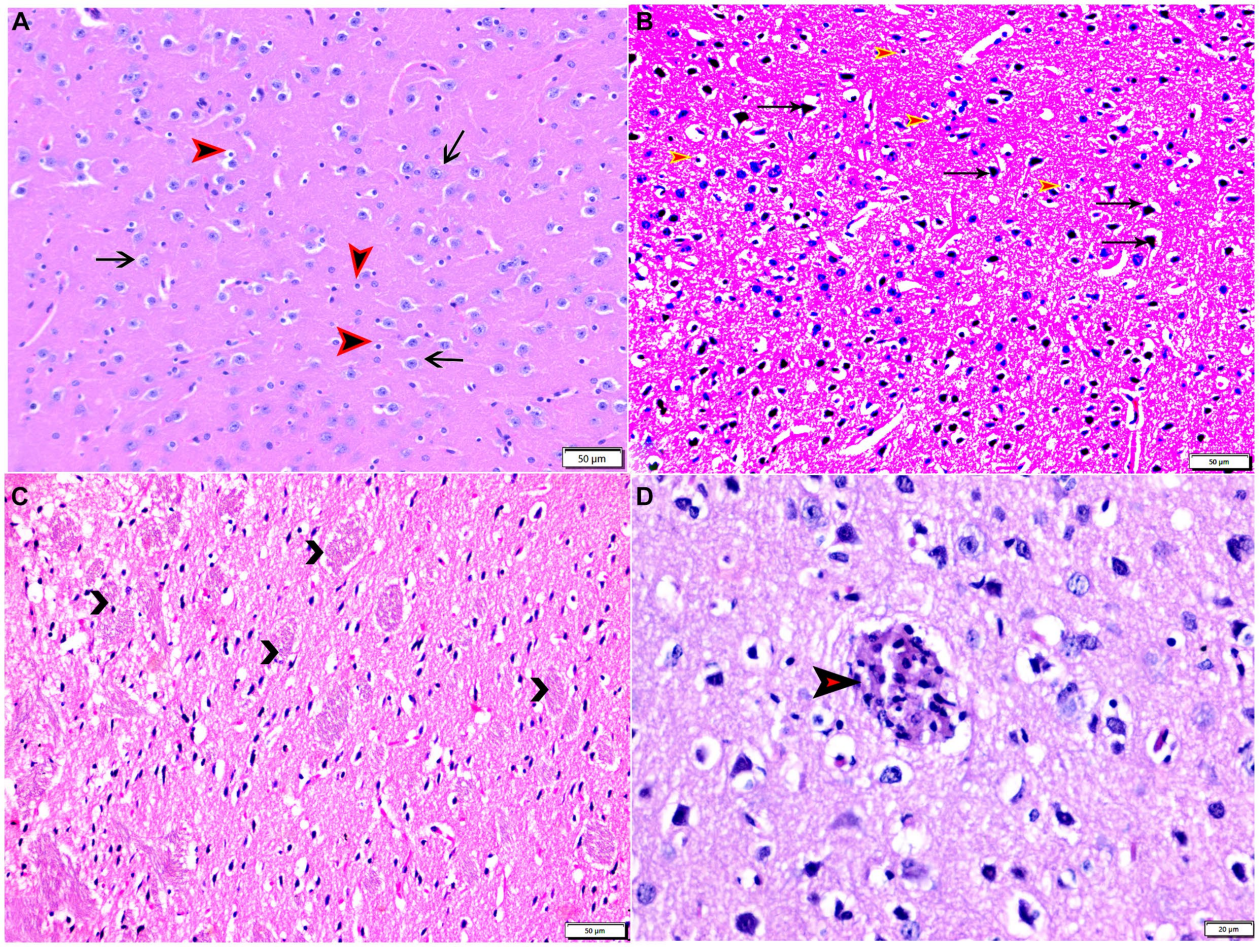
Photomicrograph of brain tissue sections from mice of experimental groups: brain tissue sections from control negative group (G1) showing **(A)** normal neuronal cells (arrows), normal glia cells (arrowheads). **(B–D)** Brain tissue sections from infected control positive group (G2) showing **(B)** marked nerve cell degeneration (arrows), marked astroglia cell reaction (red arrowheads). **(C)** Degenerated bundles of nerve fibers (arrowheads). **(D)** Parasitic tissue cyst contains multiple bradyzoites (arrowhead). Hx&E stain, the bar size was indicated under pictures.

**Figure 4:**
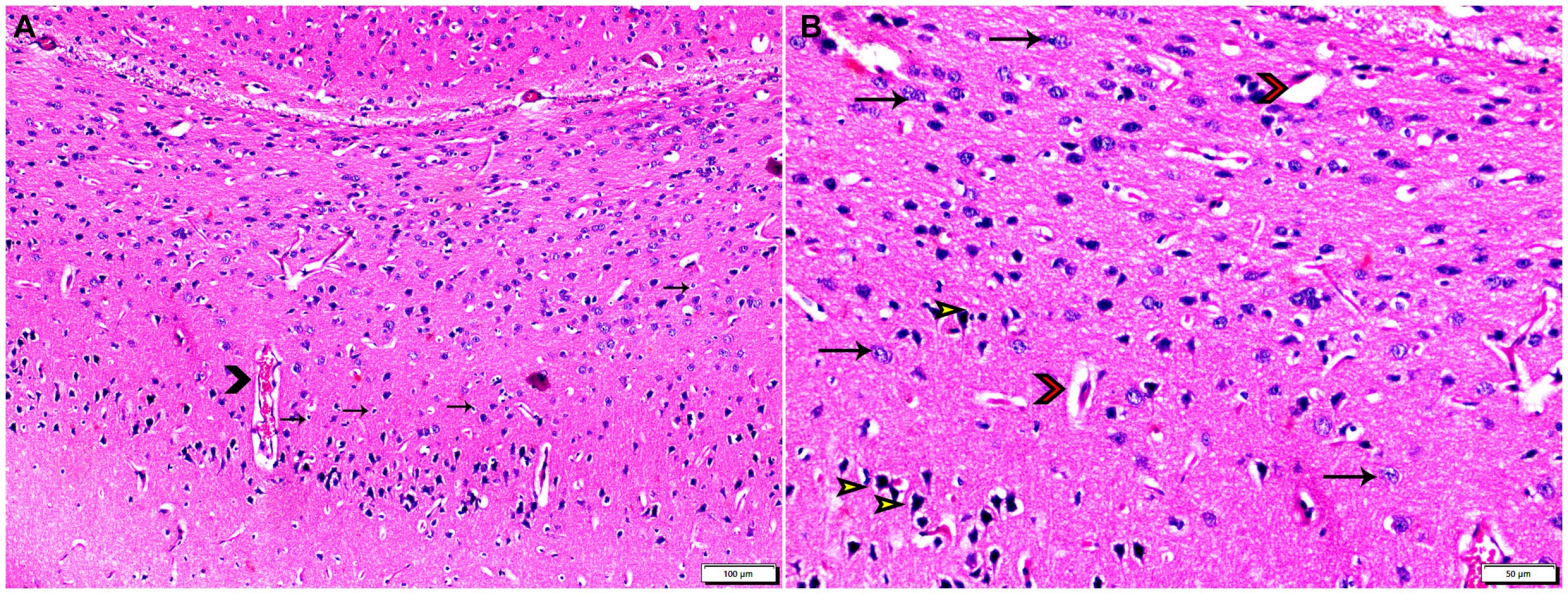
Photomicrograph of brain tissue sections from infected animals treated with propolis group (G3) showing **(A)** vascular congestion (arrowheads), mild astroglia cell reaction (thin arrows). **(B)** Capillary congestion with peri-capillary edema (red arrowheads), most of nerve cell appear normal (arrows), with some degenerated neurons (yellow arrowheads). Hx&E stain, the bar size was indicated under pictures.

**Figure 5:**
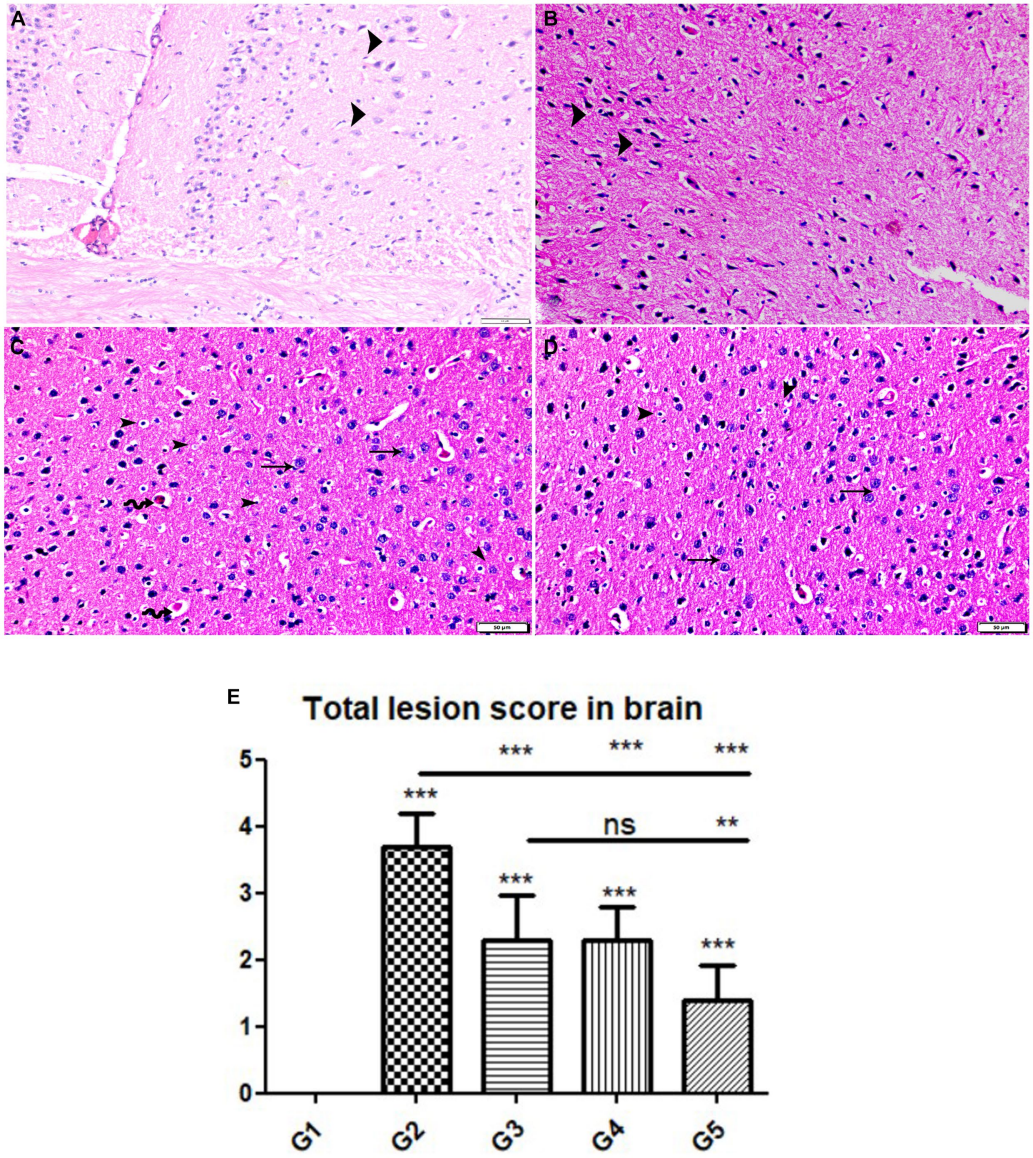
Photomicrograph of brain cerebral tissue sections from mice of experimental groups. **(A,B)** Brain tissue sections from infected animals treated with wheat germ oil (G4) showing **(A)** normal brain structure with normal nerve cells (arrowheads), other areas showing **(B)** degenerate nerve cells (arrowheads). **(C,D)** Brain tissue sections from infected animals treated with a combination of propolis + wheat germ oil (G5) marked improvement in histological structure of brain tissue except for presence of mild pericapillary edema (zigzag arrows), mild glia cell reaction (arrowheads), and normal nerve cells (thin arrows). note: absence of bradyzoites or tissue cyst. Hx&E stain, the bar size was indicated under pictures. **(E)** Histomorphometry graph showing quantitative and semiquantitative measurements of total lesion scores recorded in brain tissue sections among the experimental groups. Data are expressed as means ± standard deviations. Significant differences vs. the control group are marked by different asterisks through one-way ANOVA with Tukey’s *post-hoc* test: *p ≤ 0.05, **p ≤ 0.01, ***p ≤ 0.001.

**Figure 6:**
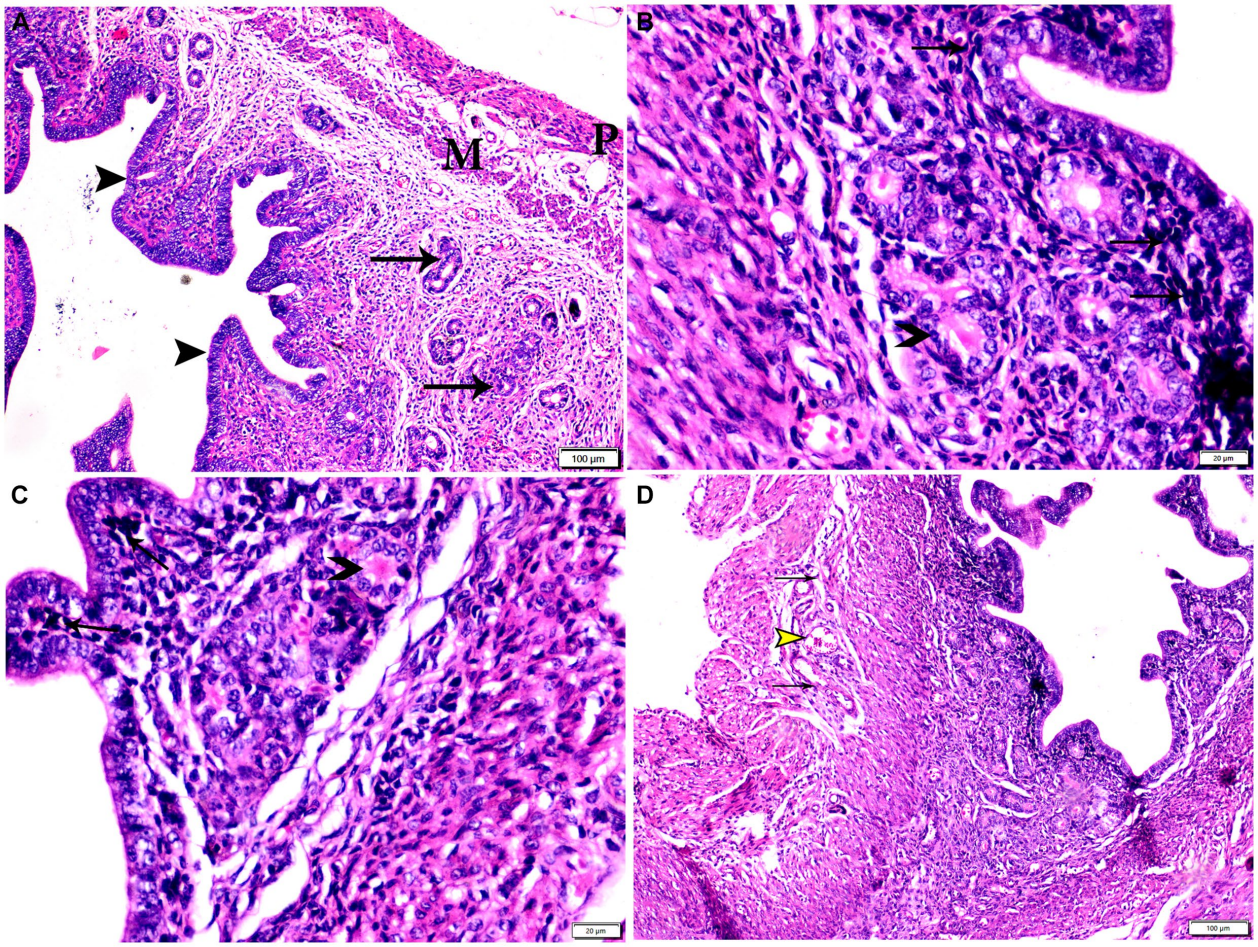
Photomicrograph of uterine tissue sections from mice of experimental groups. **(A)** Uterine tissue sections from the control negative group (G1) showing Normal endometrial epithelium (arrowheads). Normal endometrial glands (arrows), myometrium (M), and perimetrium structure (P). **(B–D)** Uterine tissue sections from the infected control positive group (G2) showing **(B,C)** multiple bradyzoites present in lamina propria of endometrium (arrows), degenerated endometrial gland (arrowheads). **(D)** Vascular congestion (yellow arrowhead) with inflammatory cellular infiltration (arrows) in myometrium. Hx&E stain, the bar size was indicated under pictures.

**Figure 7:**
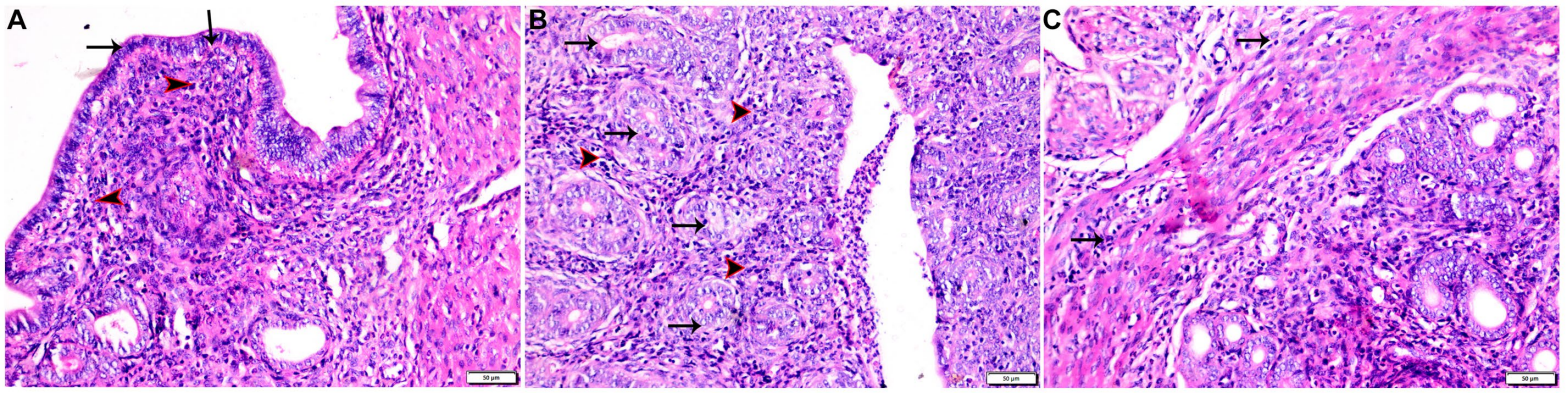
Photomicrograph of uterine tissue sections from infected animals treated with Propolis (G3) showing **(A)** normal endometrial epithelium (arrows), marked inflammatory cellular infiltration sub-endometrium (arrowhead). **(B)** Degenerated endometrial gland (arrows) surrounded by inflammatory cellular infiltration (arrowheads). **(C)** Inflammatory infiltration in myometrium (arrows). Hx&E stain, the bar size was indicated under pictures.

**Figure 8:**
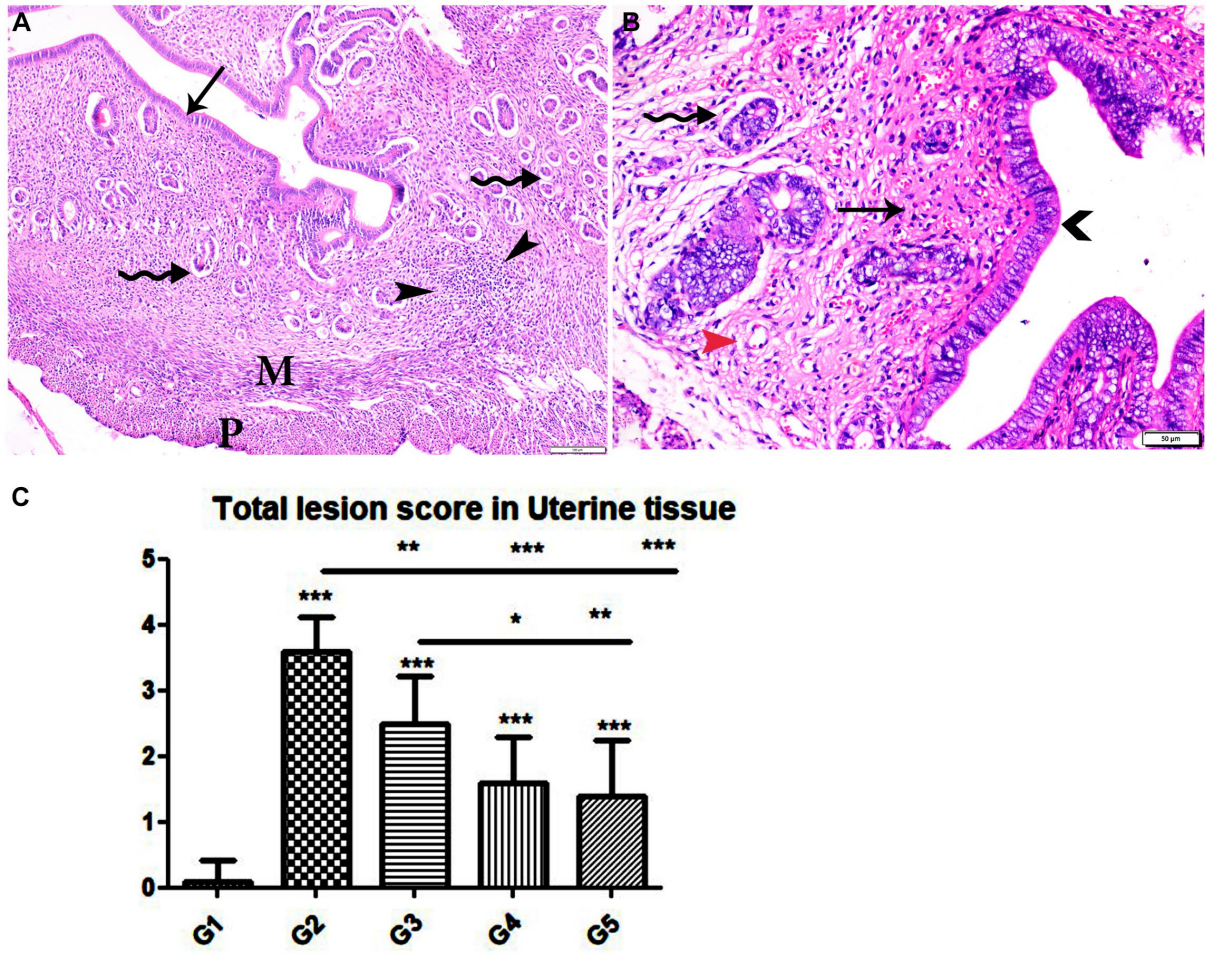
Photomicrograph of uterine tissue sections from mice of experimental groups. **(A,B)** Uterine tissue sections from infected animals treated with wheat germ oil showing **(A)** normal endometrial epithelium (arrow)., focal mononuclear inflammatory cellular infiltration in endometrium around endometrial glands (arrowheads). A atrophy and degeneration of endometrial glands with peri-glandular edema (zigzag arrows), normal myometrium (M), and normal perimetrium structure (P). **(B)** Uterine tissue sections from infected animals treated with a combination of propolis + wheat germ oil (G5): marked improvement in the uterine cellular structure, normal endometrial epithelium (arrowhead), normal endometrial gland (zigzag arrow), and mild inflammatory cellular infiltration (thin arrow), and normal blood vessels (red arrowhead). H&E stain, the bar size was indicated under pictures. **(C)** Histomorphometry graph showing quantitative and semiquantitative measurements of total lesion scores recorded in Uterine tissue sections among the experimental groups. Significant differences vs. the control group are marked by different asterisks through one-way ANOVA with Tukey’s *post-hoc* test: *p ≤ 0.05, **p ≤ 0.01, ***p ≤ 0.001.

**Figure 9:**
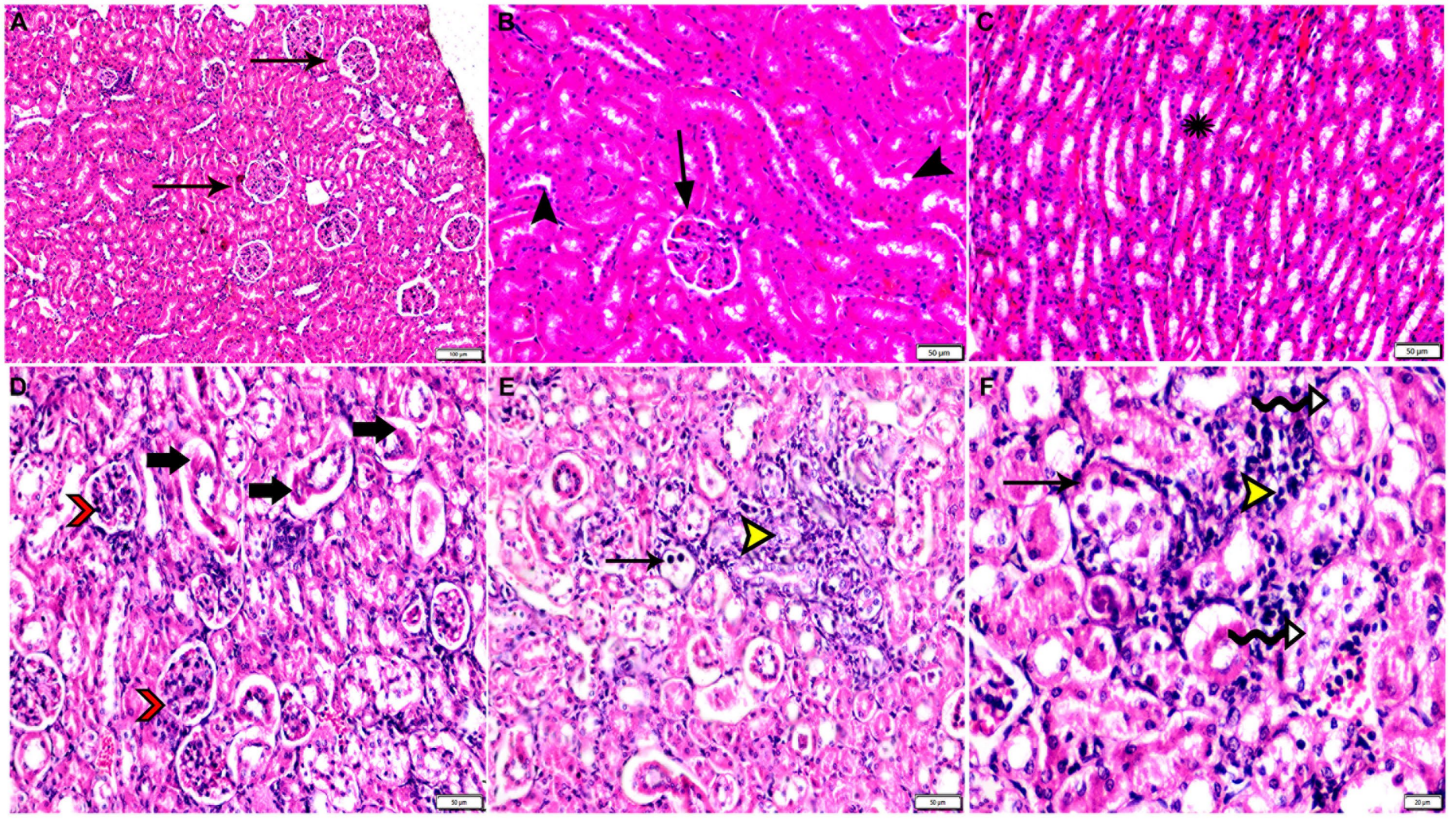
Photomicrograph of kidney tissue sections from mice of experimental groups. **(A–C)** kidney tissue sections from the control negative group showing **(A,B)** normal glomerular size and structure (arrows), normal renal cortical tubules (arrowheads). **(C)** Normal renal medullary tubules (star). **(D–F)** Kidney tissue sections from mice of infected control positive group showing **(D)** glomerular atrophy (arrowheads), degeneration and necrosis of renal tubules (arrows). **(E,F)** Multiple parasitic tissue cysts contain multiple bradyzoite (thin arrows), sever degeneration and desquamation of renal tubules (zigzag arrows). Focal mononuclear cellular aggregation (yellow arrowheads). Hx&E stain, the bar size was indicated under pictures.

**Figure 10:**
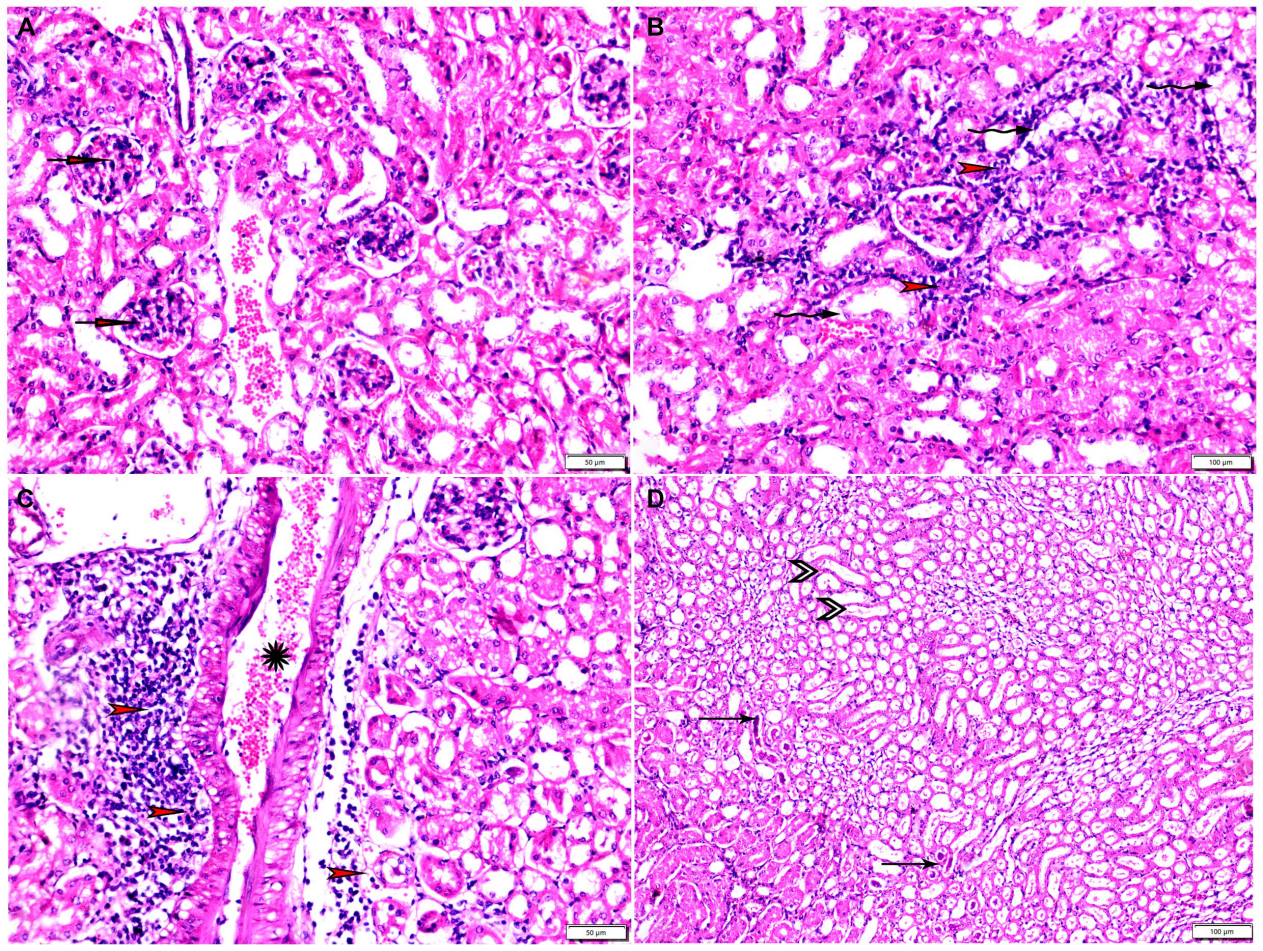
Photomicrograph of kidney tissue sections from *Toxoplasma Gondii* infected animals treated with propolis group (G3) showing **(A)** glomerular atrophy with intra-glomerular congestion (arrows). **(B)** Cortical tubular vacuolar degeneration (zigzag arrows) surrounded by sever mononuclear cellular infiltration (red arrowheads). **(C)** Sever vascular congestion (star) surrounded by mononuclear cellular infiltration (red arrowheads). **(D)** Renal medulla showing cystic tubular dilatation (arrowheads), renal cast at some tubes (thin arrows). Hx&E stain, the bar size was indicated under pictures.

**Figure 11:**
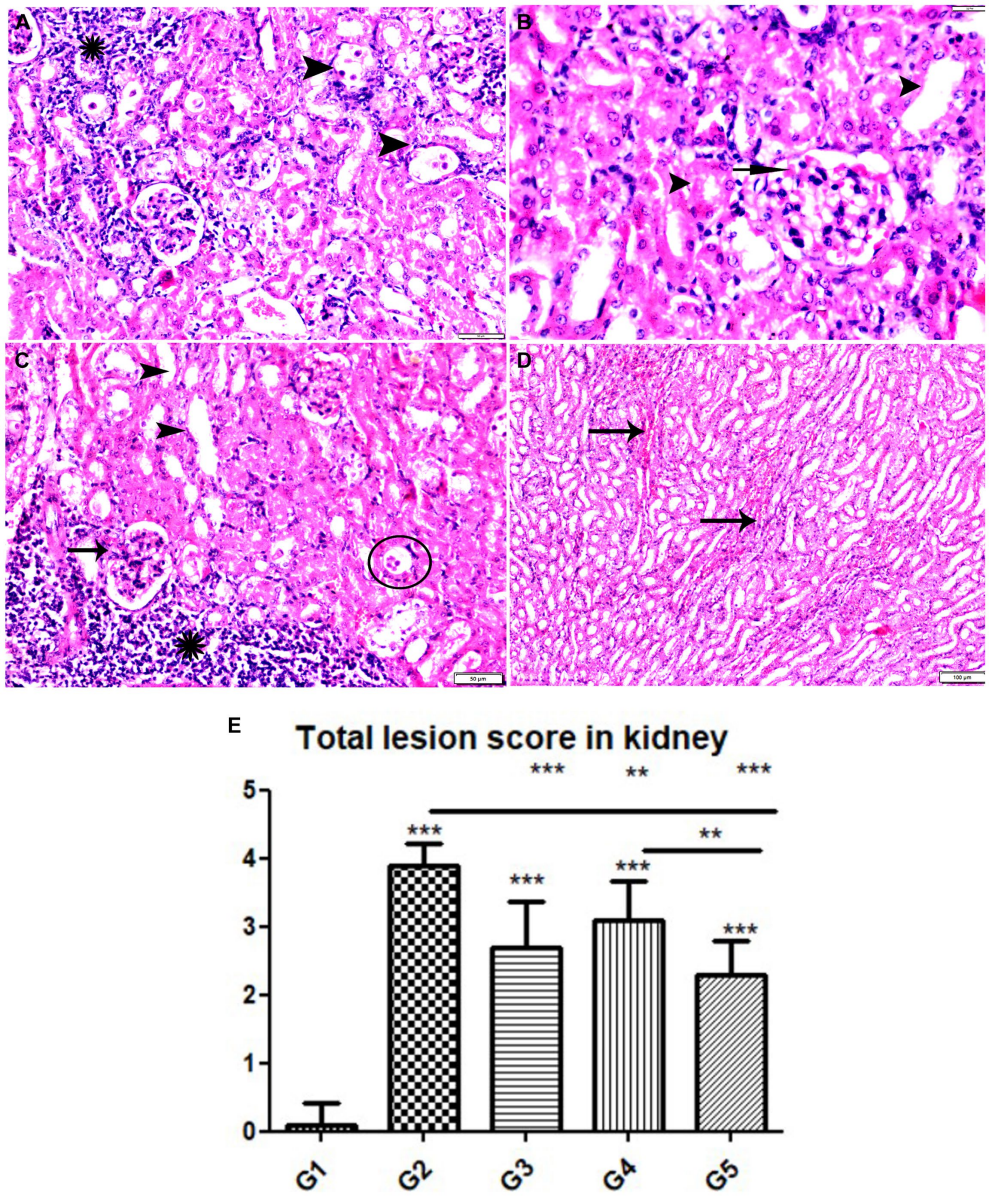
Photomicrograph of kidney tissue sections from mice of experimental groups. **(A,B)** Kidney tissue sections from mice of infected animals treated with wheat germ oil (G4) showing **(A)** tissue cysts contain multiple bradyzoites (arrowheads), aggregated mononuclear cellular inflammatory cells (star). **(B)** Normal renal tubules (arrowheads), intra-glomerular congestion (arrow). **(C,D)** Kidney tissue sections from infected animals treated with a combination of propolis + wheat germ oil (G5). **(C)** Improvement in renal cortical tissue structure: Normal glomerular size and structure (arrow), normal cortical renal tubules (arrow heads), parasitic tissue cyst (circle) and aggregated mononuclear cellular inflammatory cells (star) still present. **(D)** Mild intertubular congestion in between renal medullary tubules (arrows). Hx&E stain, the bar size was indicated under pictures. **(E)** Histomorphometry graph showing quantitative and semiquantitative measurements of total lesion scores recorded in kidney tissue sections among the experimental groups. Data are expressed as means ± standard deviations. Significant differences vs. the control group are marked by different asterisks through one-way ANOVA with Tukey’s *post-hoc* test: *p ≤ 0.05, **p ≤ 0.01, ***p ≤ 0.001.

The histopathological examinations of brain, uterus, and kidney tissues were performed and illustrated in Figures 3–11. Brain tissues of negative control healthy mice (G1) showed normal histological architecture with normal neuronal and glia cells as shown in Figure 3A. The positive control brain tissues showed different pathological alterations because of the *T. gondii* infection including astroglia cells proliferation, vascular dilatation, and perivascular edema with presence of marked nerve cells and nerve fiber degeneration as present in Figures 3B,C. In addition, many tissue cysts were detected revealing the parasitic infection as shown in Figure 3D. Most of nerve cells appeared normal with few vascular congestion, mild astroglia cell reaction, capillary congestion with peri-capillary edema in the brain tissues of mice treated with Propolis (G3) as shown in Figures 4A,B. While the brain tissues from the mice treated with WGO (G4) showed mild neuronal degeneration with mostly normal neurons and very few cysts were noticed as shown in Figures 5A,B. A marked improvement in the histological structure of the brain tissue was observed after treating mice with combination of propolis and WGO in G5 except for presence of mild pericapillary edema and mild glia cell reaction with absence of bradyzoites or tissue cyst as shown in Figures 5C,D. In consistency with the mentioned changes, histopathological scoring showed significant improvement in the treated groups (G3-5) when compared to the positive control group (G2) as shown in Figure 5E.

Specimens from uterine tissues of negative control group showed normal uterine histological architecture as shown in Figure 6A. In contrast, the inspected uterine specimens of positive control group showed degenerated endometrial epithelium and endometrial gland, inflammatory cellular infiltration in the myometrium, and several cysts were observed in the lamina propria of the endometrium as shown in Figures 6B–D. Meanwhile, the group treated with Propolis (G3) showed normal endometrial epithelium with marked inflammatory cellular infiltration in sub-endometrium as shown in Figure 7A. Also, G3 showed degenerated endometrial gland surrounded by inflammatory cellular infiltration and inflammatory infiltration in myometrium as shown in Figures 7B,C. In the group treated with WGO (G4), normal endometrial epithelium with mononuclear inflammatory cellular infiltration in endometrium around endometrial glands, atrophy, and degeneration of endometrial glands with peri-glandular edema, and normal myometrium structure were observed as shown in Figure 8A. Marked improvement in the uterine cellular structure with normal endometrial epithelium and endometrial glands were observed in the mice treated with a combination of propolis and WGO (G5) as shown in Figure 8B. In consistence with these microscopical findings, histopathological scoring showed significant improvement in the treated groups (G3-5) when compared to the positive control group (G2) as shown in Figure 8C.

The histopathological examination of kidney tissues of negative control group showed the normal renal architecture as shown in Figures 9A–C. In contrast, the renal tissues of positive control mice (G2) showed glomerular atrophy, degeneration, and necrosis of renal tubules with presence of multiple parasitic tissue cysts contain multiple bradyzoite, sever degeneration and desquamation of renal tubules was also observed as shown in Figures 9D–F. Renal tissues of mice treated with Propolis (G3) showed glomerular atrophy with intra-glomerular congestion, cortical tubular vacuolar degeneration surrounded by sever mononuclear cellular infiltration, sever vascular congestion, and renal medulla showed cystic tubular dilatation as shown in Figures 10A–D. Also, renal tissues of mice treated with WGO (G4) showed marked improvement in the whole renal structure; tissue cysts contain multiple bradyzoites with aggregated mononuclear cellular inflammatory cells were observed as shown in Figures 11A,B. Interestingly, the renal tissues of mice treated with combination of propolis and WGO were with normal glomerulus and tubular structure as shown in Figures 11C,D. Regarding the histopathological scoring, it showed significant improvement in the renal tissues of all treated groups (G3-5) when compared to the positive control group (G2) as shown in Figure 11E.

## 4 Discussion

The common drugs of toxoplasmosis are effective against the tachyzoites stage which express the acute infections, however that, it affects the vital parameters including blood count, hepatic, and renal markers, and possess different side effects specially in the immunocompromised patients (9). Therefore, plant-based antiparasitic therapy is a good strategy to avoid such alterations and side effects. In this study, we targeted the development of a plant-based therapy against the acute *T. gondii* infection to get the antiparasitic effect with no side effects of the available chemical drugs. To achieve this objective, this study investigated the effect of treating with propolis and WGO separately and in combination against acute toxoplasmosis in mice.

The current findings revealed a significant decrease in the parasitic cysts load in brain tissues after treatment with propolis in G3, as compared to the positive control infected group (G2). These findings agree with a previous research that concluded the ability of propolis to reduce the tissue bradyzoites in rats after chronic toxoplasmosis infection (31–33). The antiparasitic activity of propolis is mostly related to its ability to hinder the invasion of host cells by the parasite, via blocking the required enzymatic activity of this process (49). Moreover, propolis can upregulate the innate immune response, anti-inflammatory and antioxidant signaling (49), making it a potent antiparasitic agent in this study.

In addition, we have also investigated the antiparasitic role of WGO because of its known anti-inflammatory and antioxidant effects (27). The current findings revealed a significant decrease in the parasite load brain tissues of mice treated with WGO (G4), as compared to the control positive group (G2), and the Propolis-treated group (G3). The antiparasitic activity of WGO was reported in a previous study against chronic toxoplasmosis (32, 33) and in a previous *in vitro* study against the *Trichomonas vaginalis*, compared to the metronidazole treatment (50). It is noteworthy to state that toxoplasmosis inhibits the cytokines production from the host cells which activate the immune response, resulting in immunodeficiency (51). Properly, the antiparasitic effect of WGO in this study might be related to its ability to improve the immunity of host cells. Furthermore, WGO enhances the activated T cells, monocytes, and cytokines production revealing its immune-supportive role (52), that is why WGO could serve as a potential antiparasitic plant-based agent.

Owing to the importance of molecular investigation in identification of the parasitic load in different tissues (53), we performed RT-PCR to detect and quantify the *Toxoplasma* p29 gene. This gene located in the dense granules of the parasite besides its potent prognostic value for determination the outcome and stage of the infection (54–56). In the present study, RT-PCR revealed that the control untreated animals (G2) revealed had the highest parasite load (Tables 1, 2). In stark contrast, those animals treated with the combination of propolis (G3) and WGO (G4) had the lowest parasite load, which is consistent with parasitological and histopathological findings, confirming the potential anti parasitic activity of the tested substances. Collectively quantification of the parasite burden from brain tissues of treated and control animals confirmed the parasitological findings and coherent with those reported by histopathological examination.

It is noteworthy to mention that toxoplasmosis infection exhibits different histopathological alterations in the affected tissues. In the current study, brain, uterine, and renal tissues were examined microscopically for any pathological changes. In the positive control infected group (G2), all tissues showed multiple changes including the inflammatory cellular infiltration, degenerative changes, congested vessels, perivascular edema, and with the presence of several tissue cysts, confirming the other findings of the parasitic load and molecular data, which is in harmony with several previous studies (32, 33). The toxoplasmosis-induced pathological changes were in consistence with many previous *in vivo* studies (13, 57–59). These alterations in the tissues’ architectures were approximately restored after treatment of infected mice either with propolis (G3) or WGO (G4) and were marked improved after being treated with the combination of both Propolis and WGO (G5) compared to tissues from the negative control healthy mice (G1). The protective role of the Propolis could be related to its wide range of biological activities that is enhanced by its high content of chemical compounds such as amino acids, essential oils, phenolic compounds, and flavonoids (60). While, WGO is rich in tocopherols and vitamin E that provide the WGO with high antioxidant properties in the biological tissues (61). Given the above information, the combination of both propolis and WGO in the current study had the most significant improvements against toxoplasmosis-induced pathological alterations.

## 5 Conclusion

The present study concluded that the use of both propolis and WGO together had a potential antiparasitic activity against the acute *T. gondii* infection in mice. The combination could exhibit a synergistic effect as indicated by the significant reduction in the parasitic load and restoring the molecular and histopathological changes to their normal conditions compared to the healthy group. These data would help to achieve the novel strategy to develop a plant-based antiparasitic agent, which is more effective with no side effects.

## Data availability statement

The original contributions presented in the study are included in the article, further inquiries can be directed to the corresponding author.

## Ethics statement

The animal study was approved by the guidance of the Research, Publication, and Ethics Committee of the Faculty of Medicine, Sohag University, Egypt, which complies with all relevant Egyptian legislations in publication and research. The institutional Review Board Number is Sohag-10-7-2022-2. The study was conducted in accordance with the local legislation and institutional requirements.

## Author contributions

EE: Conceptualization, Formal analysis, Funding acquisition, Investigation, Methodology, Project administration, Resources, Software, Supervision, Validation, Writing – original draft, Writing – review & editing. FA: Conceptualization, Formal analysis, Methodology, Resources, Software, Validation, Writing – original draft, Writing – review & editing. ER-Á: Data curation, Formal analysis, Funding acquisition, Investigation, Methodology, Resources, Software, Validation, Writing – original draft, Writing – review & editing. AF: Conceptualization, Formal analysis, Investigation, Resources, Supervision, Validation, Writing – original draft, Writing – review & editing. KA: Conceptualization, Formal analysis, Methodology, Software, Supervision, Visualization, Writing – original draft, Writing – review & editing. HE: Conceptualization, Formal analysis, Funding acquisition, Investigation, Methodology, Software, Supervision, Writing – original draft, Writing – review & editing. ME-K: Conceptualization, Software, Supervision, Validation, Visualization, Writing – original draft, Writing – review & editing. ASS: Data curation, Software, Supervision, Validation, Visualization, Writing – original draft, Writing – review & editing. AHS: Data curation, Formal analysis, Software, Supervision, Validation, Visualization, Writing – original draft, Writing – review & editing. ASA: Conceptualization, Data curation, Formal analysis, Resources, Software, Supervision, Validation, Visualization, Writing – review & editing. AAS: Data curation, Formal analysis, Investigation, Software, Supervision, Validation, Visualization, Writing – review & editing. AAA: Data curation, Formal analysis, Investigation, Software, Supervision, Validation, Visualization, Writing – review & editing. AhA: Conceptualization, Data curation, Formal analysis, Funding acquisition, Software, Supervision, Validation, Visualization, Writing – original draft, Writing – review & editing. AB: Conceptualization, Data curation, Formal analysis, Investigation, Methodology, Project administration, Resources, Software, Supervision, Validation, Writing – original draft, Writing – review & editing. AM: Data curation, Formal analysis, Software, Supervision, Validation, Writing – review & editing.
